# Book Reviews

**Published:** 1877-05

**Authors:** 


### Reviews and Book Notices.

A PRACTICAL TREATISE ON MATERIA MEDICA AND THERAPEUTICS. By *Roberts Bartholow, A. M., M. D.*, Professor of the Theory and Practice of Medicine, and of Clinical Medicine, and formerly Professor of Materia Medica and Therapeutics, in the Medical College of Ohio, etc., etc. New York: *D. Appleton & Co.* 1876. pp. 537.

In his preface, as reasons for presenting to the profession a new work on Materia Medica and Therapeutics, Prof. Bartholow claims as follows:

1. A simpler classification.
2. A consideration of several new remedies, not generally referred to in works of like character.
3. A condensed treatment of individual remedies.
4. A condensed description of physiological actions, most consonant with known facts.

These are reasons good and true, apart from the arrogation of "twenty-two years of clinical experience," and "many independent investigations" contributing "some original knowledge to the subject of Therapeutics."

Let us, by examination, see how far his work sustains his claims.

I. A simpler classification.

Prof. Bartholow's classification is as follows:

*Part* 1. Modes in which medicines are introduced into the organism.

*Part* 2. The actions and uses of Remedial Agents.

Those used to promote constructive metamorphosis.

Those used to promote destructive metamorphosis.

Those used to modify the functions of the nervous system.

Those used to cause some evacuation from the body.

*Part* 3. Topical remedies.

This is simple enough, most certainly.

Under *Part* 1 are included, applications through the *external integument;* the *internal integument*, viz., the broncho-pulmonary, gastro-intestinal, and genito-urinary mucous membranes; the *subcutaneous areolar tissue;* and the *veins.*

In *Part* 2, under *Remedies used to promote constructive metamorphosis*, are Aliments, Water and Hydrotherapy, Pepsin and Lactic Acid, Mineral Acids, Oils and Fats, Mineral Tonics, and Vegetable Bitters, with their Alkaloids.

Under *Remedies used to promote destructive metamorphosis*, are Alkalies, Ammonium and its preparations, Alteratives, (so-called) Metals (as Lead, Copper, etc.), Tannic Acid, and vegetable astringents.

Under *Remedies used to modify the functions of the nervous system*, are Excito Motors, Cerebral Excitants, Cerebral Sedatives, and Motor Depressants.

Under *Evacuants* are Emetics, Cathartics, Anthelmintics, and Urino-Genitals.

*Part* 3 contains Antiseptics, Counter Irritants, Epispastics, Blood-letting, Escharotics, Emollients, Demulcents, and Protective Agents.

Between the cumbersome classification of Prof. George B. Wood, and the no classification at all of Dr. Sidney Ringer, Prof. Bartholow most certainly strikes a very happy medium. Though one may question the propriety of placing tannin among agents producing waste, and blood-letting among topical remedies, still any classification must be deficient, with our limited knowledge of the physiological actions of many remedies. The supposition is rational, that the simplest classification must therefore embody the least error.

II. A consideration of new remedies, not generally referred to.

If, by new remedies, Prof. Bartholow refers to agents recently introduced, and now undergoing the test of clinical usage, he may be credited with good articles on Apomorphia, Eucalyptus, Jaborandi, Croton Chloral, Salicylic Acid, and Salicin.

If, however, his reference is to agents not found in other recent works on Therapeutics, his claim is invalid, since both Sidney Ringer and H. C. Wood write fully on the above agents, and certain Botanic remedies, such as Hamamelis, Sabbatia, Alnus Serratula, and others are found in the lists of other schools.

His book, however, contains articles on several subjects not usually treated of in works on Therapeutics; viz., An elaborate article on Alimentation, considering foods, animal and vegetable, special plans of diet and alimentation in disease; a full list of Chalybeates, Saline, Alkaline, and Sulphurous Mineral Waters; a clear and concise article on the kinds and uses of Electricity; excellent directions for and against the use of Anesthetics; and good but brief articles on the combined use of Morphia and Atropia, and the theory of counter irritation.

III. A condensed treatment of individual agents. This claim is most fully substantiated.

The method of treatment is excellent, and as follows:

1. The Drug with English, French, and German synonyms.
2. Its various preparations with doses.
3. Antagonists and Incompatables.
4. Synergists; viz., Remedies which assist its action.
5. Physiological Actions.
6. Therapy.
7. Authorities referred to.

Each article also is wonderfully condensed and simplified by excluding all botanical, chemical, and pharmaceutical details, except so far as to explain compound preparations or incompatability. This seems to us proper and advantageous. There is no more reason why such details should enter a work devoted mainly to therapeutics, than that descriptive anatomy should be bound up with a work on surgery. Among "various preparations" are included all popular or valuable ones that are unofficinal, with frequent directions for their combination. The lists of synergists are also valuable and instructive. The sections on physiological actions are brief, and to the point; while those on therapy include all established uses of the several remedies, whether suggested by physiological actions or obtained from long clinical experience. Scattered throughout each section on therapy are many formulæ drawn from his own experience, or that of men eminent in the profession. An excellent feature of this method is the placing of authorities quoted, in a list at the end of each article, rather than in foot notes, or in brackets on the pages.

IV. The fourth claim; viz., a condensed description of physiological actions, most consonant with known facts, is equally well maintained.

Whoever has read the recent able and scientific work on Therapeutics, by Prof. H. C. Wood, must, at times, have been thoroughly confused by the conflicting physiological actions assigned by eminent authorities, to the same remedy. This has been avoided by Prof. Bartholow. Where physiological actions are well established, they are briefly given. Where there is uncertainty, the doubt is stated, and the best authenticated actions are given as the probable ones. Much confusion and needless discussion is thus avoided.

If there is a weak element in the work under discussion, it lies in the empirical basis of much of the therapy. To use Prof. Bartholow's words; "Although convinced that the most certain acquisitions to therapeutical knowledge must come through the physiological method, I am equally clear that well established empirical facts should not be omitted, even if they are not explicable by any of the known physiological properties of the remedies under discussion."

This expressed intention has been fully carried out in his work, and detracts from its scientific character. While excuse may be found in the fact that extended physiological experiment is daily establishing many clinical experiences, still it is equally true that a popular empirical fact of to-day may become a fiction to-morrow. A graver objection, however, rests in the license it gives the author to state personal theories and beliefs. It is creditable to Prof. Bartholow that he has availed himself so little of the opportunity.

To sum up—Prof. Bartholow has given to the profession a valuable work on Therapeutics—and one bound to be popular. Its excellencies consist in a very simple yet sufficient classification—the exclusion of all unnecessary details—a clear, concise, readable style—a most excellent method of treating individual subjects—and a practical therapy. As the "learned and encyclopædic volumes" of Stillé are best suited for reference, and the scientific work of H. C. Wood is the best exponent of the physiological research of the day, so is the practical book of Bartholow the best hand-book for the medical student and busy practitioner. J. S. K.

TEXT-BOOK OF HUMAN PHYSIOLOGY—DESIGNED FOR THE USE OF PRACTITIONERS AND STUDENTS OF MEDICINE. By *Austin Flint, Jr., M. D. D. Appleton & Co.*, 1876.

This book is of too high an order to be written upon after a mere cursory examination. This is our only apology for so late a review.

The opening chapter is devoted to the consideration of the blood, seemingly in accordance with that old theory, "the blood is the life," which becomes more and more true in the light of modern science. The exposition is worthy of the subject; we can take time for the disputed questions only. The first we notice is in regard to the existence of a nucleus in the red corpuscle, which the author denies, but which we believe is acknowledged by some very good authorities—though we ourselves have always considered the so-called nucleus simply an optical effect. There seems, however, to be sufficient reason to consider the question as still an open one.

The next point is in reference to the development of the corpuscle, and the radical view the author takes of this is in great contrast to the conservative views which he takes of certain other subjects. The theory of the development of the red from the white corpuscles, is unequivocally denied. "They appear by genesis in the sanguineous blastema," is the author's language. As to the leucocytes, the experiments of Onimus are cited to prove that the "corpuscles may be developed under *favorable circumstances* in a perfectly clear homogenous blastema." The italics are our own, for it is upon these "favorable circumstances" that the whole question hinges. What are they? First, the liquid was placed under the skin of a *living* animal. If the corpuscles are developed in the blastema, de novo, why not secure for it, the blastema, the same temperature and the same osmotic action, both purely mechanical, entirely *away from* a living animal. We must confess this getting so close to a living animal, looks very suspicious on the part of "spontaneous generation."

Second, when *too thick* an animal membrane was used, or when the same blastema was envoloped in a sac of caoutchouc or in a glass tube hermetically sealed, there was no development of leucocytes. "From this it was concluded that osmotic action is a necessary condition, and that the mere heat of the body is not sufficient to develop these corpuscles, even in an appropriate blastema." Our version would be this; from this it was concluded that the membrane must be sufficiently porous to admit either cells or the germs of cells, which abound in the connective tissue and lymphatics just beneath the skin of any living animal, that possesses a skin. In order to be conclusive, the conditions of the experiment should preclude the possibility of the entrance of germs, but we observe that whenever such possibilities were precluded, no cells were formed.

The theories in regard to the constitution of the blood plasma are fairly discussed; that of Denis is the one adopted, and is altogether the most plausible; viz., that the blood liquor is composed of plasmine and serine. The decomposition of plasmine results in coagulable fibrin and dissolved fibrin or metalbumen. This does away with the necessity of admitting the existence of paraglobulin (fibrinoplasm) and a ferment —fibrinogen, as proposed by Schmidt and adopted by Virchow and others. The other constituent serine is simply osmotic albumen, probably corresponds to albumenose, a diffusible albumen.

The chapter on the heart is most excellent. The diagrams are especially fine—from Bomany and Beace. The influence of respiration on the action of the heart, is not explained as fully as might be. The fact that during expiration the internal pressure is greater than the external, whereby the pressure upon the heart and its great vessels is correspondingly increased, is not even suggested.

The cause of arrested circulation in asphyxia is said to be mechanical; "unaerated blood cannot circulate in the systemic capillaries." We believe it has been demonstrated by direct experiment, that black blood circulates as well as red—the cause of the stasis is not in the action of the venous blood upon the vessels or heart directly, but indirectly through the nerve centres, whose function is arrested by this venous blood. This is the theory of Bichat, and is certainly the most reasonable. As to the cause of the rythmical contraction, the author very truly calls it an inherent property of the muscular fibre. The function of the nerve of Cyon and the co-operation of the splanchnic with that nerve are very lucidly explained.

In the causes of respiration, the very cause of causes—inequality of pressure—is not mentioned, whereas the whole mechanical apparatus of respiration, is subservient to the production of this inequality.

The chapter on alimentation is full, and we are glad to see that the author takes no equivocal position on the alcohol question. "Under ordinary conditions, where the organism can be adequately supplied with food, alcohol is undoubtedly injurious. * * * The effects of its continued use, conjoined with insufficient nourishment, show that it cannot take the place of assimilable matter." While Dr. Hayes' testimony cannot be disputed that "strong, able-bodied men have become utterly incapable of resisting cold in consequence of the long-continued use of alcoholic drinks."

The subject of digestion is amply considered, the author deciding that it is lactic and not hydrochloric acid that is found in the gastric juice. In the syllabus of this chapter we find this heading: "Action of the gastric juice upon the coats of the stomach," but we find nothing on this subject in the text.

The physiological anatomy of the small intestine, is especially commendable. The drawings are from Sappey, and need no recommendation other than the name of that master anatomist. The villi and the patches of Peyer are as nearly as possible the actual objects themselves. We find no function assigned the patches, though for some years, many of the German physiologists and histologists have classified them as a part of the great lymphatic system. This whole question of ductless glands or lymphoid organs, as Frey terms them, marks one of the conservative positions of the author. Apropos of this we find further along that the vermiform appendix has no function. (?)

The action of the pancreatic juice on fats, the author, in accordance with the best authority, considers as mechanical, not chemical. It is demonstrated by Hoppe-Seyler and others, that pancreatine is composed of three separate ferments, acting respectively on the three kinds of food; and that the spleen directly influences the ferment that acts upon the albuminoids—facts not stated by Prof. Flint.

On the function of the bile, the author says this fluid has nothing to do directly with the digestion of any of the alimentary principles. Thus all the old theories are demolished, but no new one established, unless we accept that of Küss and his school, viz., that the bile renovates the mucous membrane, especially the epithelial element, which is the active agent in the absorption of fats. This theory is certainly worthy of consideration, though it is not mentioned by the author.

The most important subject in the chapter on the larger intestine is that of stercorine, or transformed cholesterine; and we may say here that the chapter on cholesterine is one of the most instructive in the whole book. Prof. Flint deserves great credit for his investigations of this subject, not only on account of its physiology, but its pathology, for he has demonstrated that the blood poisoning which follows serious structural disease of the liver, is due to this retained excretion,—cholesterine,—and has named the disease cholesteraemia.

Absorption is well discussed, especially that of the lymphatic system, though the author agrees with Sappey in disagreeing with Recklinghausen and his school, as to the origin of the lymphatics in the connective tissue by means of canaliculi or lacunæ. The attempted explanation of the absorption of fats by means of stomata in the coats of the vessels, is, it seems to us, entirely inadequate when applied to the absorption of the small intestine, for before the fat enters the vessels it enters the epithelial cells of the villi—they are found filled with fat granules during digestion. While it is probable there are stomata in the coats of vessels, it is hardly probable they exist in the walls of these cells, unless they exist as the "pore-canals" discovered by Funke and Koelliker many years ago.

The author denies the formative action of the lymphatic glands in the production of leucocytes, but it is easy to see that he is obliged to do this in order to sustain his theory of the blastema formation. As regards the application of physical laws to the function of absorption, it is of no avail as applied to the absorption of fats, and we can but agree with Longet and others, that osmosis loses its force in the presence of living elements. Indeed, the whole phenomena of secretion can be accounted for in no other way save to say that the cell has inherent power to take what it wants.

The presence of urea in the lymph in greater quantity than in the blood, demonstrated by Bernard, is very suggestive of the function of the lymphatics.

The distinction between a secretion and an excretion, made first, we believe, by Simon, is well maintained by the author, viz., "that a secretion is never discharged from the body; it has a function to perform, and it is never found in the body after the peculiar gland cells that form it are destroyed," while an excretion is just the opposite. The secretion of milk seems to be an anomaly in the mechanism of secretions, for milk, instead of being the product of glandular cells, seems to be the product of an amorphous membrane, but we venture a prophesy that this is only a seeming.

While the anatomy of the kidney is accurately given, the results of that anatomy are not followed out; indeed, the fact of increased blood pressure in the glomeruli, is mentioned only incidentally, whereas it is the great factor in the mechanism of renal excretion.

One of the most instructive divisions of this subject of the kidney, is that which treats of the influence of muscular exercise upon the composition of the urine. This embraces the substance of the author's well-known observations in the case of the pedestrian Weston, in which he found the amount of urea increased daily 15 per cent. above the average. Directly the opposite result was obtained some ten years ago by Fick and Wislicenius, but as the observation extended over one day only, the result cannot be considered reliable. As to the influence of mental exertion the author considers that no one more than another of the solid matters is increased, though many authors claim an increase of the phosphates.

The chapter on the liver is perhaps the most thoroughly scientific part of the whole book, containing much of original investigation. The double function is well demonstrated, but not so the double structure maintained by many histologists. The experiments in regard to cholesterine and glycogen, and the deductions drawn therefrom bear the stamp of true scientific spirit, and we may say here that the author's manner of summing up deductions, and his pertinent questions by which he keeps constantly before the mind just what he is searching for, makes the book of invaluable benefit to the student. While it imparts information, it imparts it in a disciplined, methodical manner.

Altogether, it is the most perfect text-book on the subject of physiology we have found in the English tongue.

S. H. S.

On Addison's Disease: Being the Croonian Lectures for 1875, delivered before the Royal College of Physicians. Revised and illustrated by plates and reports of cases. By *Edward Headlam Greenhow, M. D., F. R. S.*, etc. Philadelphia: Lindsay & Blackston. Octavo. pp. 212.

This book comprises three lectures, and, in an appendix, detailed reports of 37 cases, which in their histories support the views of the author, while in another appendix are arranged, in groups for the most useful study, 333 cases, which the author has collected as bearing upon the subject of Addison's disease.

The first lecture is devoted mainly to a careful description of Addison's disease, "together with the true characters of the pathological lesions, in and around the supra-renal capsules," found to exist in this affection. One of these lesions consists in the infiltration of the capsules by "an inflammatory exudation of a low type, which destroys the natural structure of the organs, and finally itself undergoes caseous degeneration." There is clear evidence of inflammation in the cellular envelope of the capsules, from which arise adhesions of these masses to adjacent structures, and hypertrophy of the fibrous investment of the nerves, giving the appearance of a thickening of the latter, when really their structure is not changed.

In the second lecture the author has endeavored to show "the concurrent testimony of facts in proof of the real connection subsisting between the clinical symptoms of Addison's disease and the one specific lesion in the supra-renal capsules;" and "the baseless nature of the misconceptions which have prevented the general recognition of its reality."

In the third lecture, an explanation of the etiological and pathological processes of the disease is attempted. Dr. G. believes the great increase of connective tissue in and around the capsules, compressing not only the nerves of these structures, but influencing if not seriously injuring the solar plexus and semi-lunar ganglia in the neighborhood, to be the true key to the explanation of all the symptoms. The mere destruction of the capsules and the abolition of their functions, he is sure cannot account for the phenomena.

These lectures are a scholarly and searching review and presentation of the subject. The value of the book is very greatly enhanced by the two appendices, and by the five elegant plates that illustrate the text.

Lectures on Fever: Delivered in the theatre of the Meath Hospital and County of Dublin Infirmary. By *William Stokes, M. D.*, etc. Edited by *John William Moore, M. D.*, etc. Philadelphia: H. C. Lea. 1876.

Here are 33 lectures on the subject of Fever, by an eminent man. Their delivery was spread over many years—nearly twenty; they were not delivered in any systematic order; notwithstanding careful revision, all the contradictions furnished by one lecture of the opinions expressed in another, have not been eliminated; considerable space is devoted to the discussion of the separate identity of typhus and typhoid fevers—a discussion utterly useless to an American student; they are bare of histological research, and of any study of the chemico-vital states of the fluids or the organs. While we discover in these facts serious drawbacks to the completeness and value of the work, and a reason why it will not find its way to the tables of many practitioners; the lectures contain a vast deal of the history of the study of fever, and the writings upon it, beside an exhaustive discussion of several important phases of the subject.

Report of the Fifth International Ophthalmological Congress held in New York, September, 1876. Published by a committee of *H. Knapp*, *H. D. Noyes*, *Chas. S. Bull* and *R. H. Derby*. New York: Appleton & Co. 1877.

The peculiar feature of the late *International* Congress was the great scarcity of European delegates; seven oculists were all that the old world could afford to send across the water for the occasion!

A great many papers were read; but the most of them as we naturally may expect of papers which are to be submitted to an assembly of specialists,—dwell upon very special topics and theoretical speculations, from which the physician cannot derive any practical information in regard to the management of diseases of the eye.

The question of introducing the meter-measure for the determination of lenses was brought again before the congress. This innovation has been agitated ever since 1867, and after a full discussion pro and contra at the meetings of the previous congress and in the ophthalmological journals, the project seemed to have gained the support of the leading ophthalmologists. At least, no opposing voice was heard at the congress, though no definite action was taken in the matter.

Most of the papers, being presented by American oculists, the report reflects great honor upon the scientific energy of our specialists.

The report is printed on very good paper, and *neatly bound*, an improvement which we hope the publishing committee of the future congress will adopt.

